# Study of Mechanical Behavior in Epiphyseal Fracture Treated by Reduction and Cement Injection: No Immediate Post-Operative Weight-Bearing but Only Passive and Active Mobilization Should be Advised

**DOI:** 10.3389/fbioe.2022.891940

**Published:** 2022-07-04

**Authors:** A. Moufid, P. Bokam, G. Harika-Germaneau, M. Severyns, L. Caillé, V. Valle, T. Vendeuvre, A. Germaneau

**Affiliations:** ^1^ Department of Orthopaedic Surgery and Traumatology, University Hospital, Poitiers, France; ^2^ Institut Pprime UPR 3346, CNRS—Université de Poitiers—ISAE-ENSMA, Poitiers, France; ^3^ Unité de Recherche Clinique Pierre Deniker, Centre Hospitalier Henri Laborit, Poitiers, France; ^4^ CERCA UMR 7295, CNRS—Université de Poitiers, Poitiers, France

**Keywords:** Tibial plateau fractures (TPF), PMMA cement, cancellous bone, strain distribution, balloon kyphoplasty, interface

## Abstract

The development of new percutaneous treatment techniques using a balloon for the reduction and cement for the stabilization for tibial plateau fractures (TPF) are promising. The biomechanical changes brought by the cement in the periarticular fracture are unknown. The objective of this study was to provide elements of understanding of the bone behavior in an epiphyseal fracture treated with cementoplasty and to define the modifications brought about by the presence of this cement in the bone from both an architectural and biomechanical point of view.

*In vitro* animal experimentation was conducted. Bones samples were prepared with a cavity created with or without cancellous compaction, aided by balloon expansion following the same protocol as in the treatment of TPF. A uniaxial compression test was performed with various speeds and by using Heaviside Digital Image Correlation to measure mechanical fields. Preliminary finite element models were constructed with various boundary conditions to be compared to our experimental results.

The analysis of the images permits us to obtain a representative load vs. time response, the displacement fields, and the strain distribution for crack initiation for each sample. Microcracks and discontinuity began very early at the interface bone/cement. Even when the global behavior was linear, microcracks already happened. There was no strain inside the cement. The finite element model that matched our experiments had no link between the two materials.

In this work, the use of a novel correlation process highlighted the biomechanical role of the cement inside the bone. This demonstrated that there is no load transfer between bone and cement. After the surgery, the cement behaves like a rigid body inside the cancellous bone (same as a screw or plate). The cement provides good reduction and primary stabilization (mini-invasive approach and good stress distribution), permitting the patient to undergo rehabilitation with active and passive mobilization, but no weight-bearing should be authorized while the cortical bone is not consolidated or stabilized.

## 1 Introduction

Tibial plateau fractures (TPF) are known to be arthrogenic (Honkonen, 1995; Rademakers et al., 2007). They result from high-energy injuries in young people and low-energy injuries in over-weight and elderly people (Cole et al., 2009). The gold standard treatment remains an open reduction and osteosynthesis using a plate and screws (Krause et al., 2018) which means that at the time of total knee prosthesis, the knee would have undergone at least three surgeries: the osteosynthesis, then the removal of the plate, and the prosthesis which increases the risk of infection and complication (Suzuki et al., 2011). That is why the development of new percutaneous techniques using a balloon for reduction and cement for stabilization are promising (Vendeuvre et al., 2013; Belaid et al., 2018).

PMMA cement has been used in orthopedic surgery since the 1960s (Charnley). Its initial use allowed for the sealing of joint prostheses (Smith, 2005). Since 1984, new fields of use have emerged with a standalone application in the field of traumatology in the spine, and since 2010, in different anatomical localizations (calcaneus and tibial plateau) (Vendeuvre et al., 2013; Smith, 2005; Prod’homme et al., 2018). This new approach allows minimally invasive management, reducing postoperative complications and immobilization time. The vertebral metastatic fracture were the first beneficiaries of PMMA cement in the 1980s (Onimus and Bertin, 1982). Considering the good results in the medium term, its use tends to become more democratic in some teams, even in the treatment of young patients, raising new questions: what is its role in consolidation? What tolerance is there in the long term? What biomechanical effect does it produce? Is it stable enough to permit weight-bearing? Is it stable enough to permit active and passive mobilization? What role does it play after consolidation? How does the human body react cytologically, histologically, and biomechanically to the presence of this cement initially and once the fracture has consolidated? Up to the present, no study has answered all these questions.

Animal experimentation is the only way to quantify the effects of time on the bone with cement in an organic environment after consolidation. The first step is to provide an element of understanding *in vitro* to ensure the feasibility of the *in vivo* study and to uncover a basic understanding for future *in vivo* experiments.

Measurements of the displacements and strains in bone and cement are needed to characterize and understand the biomechanical modifications that imply the presence of cement in the bone at the time of the fracture and after consolidation.

Formerly, the main source of measuring the strain in soft tissues (Larrabee, 1986; Nagarkatti et al., 2001) and hard tissues (Yang et al., 2011) was by using strain gauges and extensometers. However, gauges can be too large when compared to the scale at which strain gradients are evaluated in tissues (Nicolella et al., 2001; Väänänen et al., 2013). The extensometers sometimes can damage the bone. When the computational side is considered, particularly finite element (FE) analysis, there was a total reliance on experimental data as an input and result validation. Therefore, the alternative is optical measurement techniques such as holographic interferometry and speckle interferometry although they are quite sensitive to minute displacement fields. In such a predicament, digital image correlation (DIC) (Schreier et al., 2009) turned out to be an assuring optical technique in the biomechanical field (Bay, 1995; Bay et al., 1999). However, there are some limitations such as lack of accuracy and precision due to correlation errors, substantial noise, out-of-plane movements of the surface during testing, and uncertainty problems in the vicinity of the crack junction. An improved DIC method (Heaviside-based DIC) was developed and used on bone tissue to compensate for these limitations (Valle et al., 2015). In addition, finite element methods were used recently to study the microdamage in the bone distribution in the cancellous bone and bone interface (Tozzi et al., 2012; Srinivasan et al., 2017). However, no works so far have reported on the mechanical understanding of the bone–cement interface in the field of traumatology.

Therefore, the objective of this study was to provide elements of understanding of the bone behavior on an epiphyseal fracture treated with cementoplasty to measure the mechanical effects of cement injection on adjacent bone structures and to know if it would be possible to advise weight bearing or simple passive and active mobilization postoperatively. For that, we had to define the modifications brought about by the presence of this cement in the bone under compressive stress from both an architectural and biomechanical point of view using Heaviside-based DIC to evaluate displacement fields and identification of multiple cracks.

## 2 Materials and Methods

### 2.1 Specimen Preparation

Metaphyso-epiphyseal bone from a 6-month calf knee was used. The bone samples were cleaned of flesh and tissues. Metaphyseal regions with a good amount of cancellous bone were chosen. We reproduced the protocol of preparation and injection identical to that used to treat the cortico-spongious fracture. The first step was to dig a hole with a square tip, and then two kinds of samples were prepared. In three samples, a balloon was inflated (2ml and 500PSI of pressure), then the created cavity was filled with PMMA cement with a texture similar to the one used in the surgical treatment of the fracture. In three other samples, the cavity was just enlarged using a drill bit of 6 mm and then filled the same way the samples with the balloon were. Cubic samples were extracted using a mechanical saw with the dimension of 15*15*10mm^3^.

**FIGURE 1:**
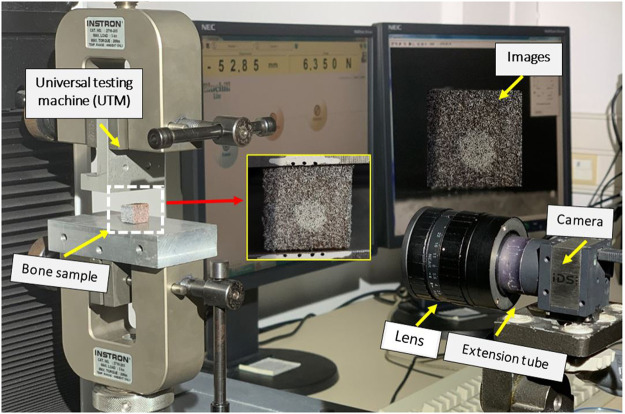
Uniaxial compression test with speckle pattern on bone sample and markers on the support.

Samples were preserved from degradation and stored in a refrigerator (−20°C). Before mechanical testing and before cement injection, the samples were washed with normal saline commixed with ethanol and dried in an oven at approximately 40°C to abstract moisture content (Voor et al., 2004). A speckle pattern was made on all samples using paint spray as shown in Figure 1.

### 2.2 Mechanical Experiment

The mark-tracking technique was used to measure imposed strain from markers deposed on the supports precisely. The surface of the specimen was colored by spraying white spots on a black opaque layer to increase the contrast and create a random distribution of gray levels (Figure 1). Uniaxial compression testing was performed using an Instron universal testing machine (5 kN). The compression tests were conducted under displacement control at three different strain rates (Shim et al., 2005) (Table 1) until cracks or damage in the sample were observed.

**TABLE 1:** Strain rates used for compression tests.

	Strain rate (mm/min)		
Sample with balloon	0.001	0.05	0.1
Sample without balloon	0.001	0.05	0.1

Continuous images were recorded every 2 s during the compression test (Figure 1). The loading stopped when the curve dropped from the maximum load.

### 2.3 Heaviside Digital Image Correlation (H-DIC)

In the present work, we use a novel technique (Heaviside DIC) based on Digital Image Correlation, adapted to measure the presence of cracks. This method was described in a previous article with an application in rock mechanics (Valle et al., 2015) or analysis of cracks in bone tissue (Bokam et al., 2020). H-DIC is an extension of the classic DIC method, for which we recall that the correlation function S is described as Eq. (1)

$$S\left(u,v,\;\frac{\partial u}{\partial x},\;\frac{\partial u}{\partial y}\;,\;\frac{\partial v}{\partial x},\;\frac{\partial v}{\partial y}\right),$$ (1)

and the kinematical transformation is defined [Eq. (2)] by simple in-plane translations $\;\underline{U}=\left(u,v\right)\;$ and the first gradients Eq. (2)

$$\begin{array}{l} \frac{\partial \underline{U}}{\partial \underline{X}}=\left(\;\frac{\partial u}{\partial x},\;\frac{\partial u}{\partial y}\;,\;\frac{\partial v}{\partial x},\;\frac{\partial v}{\partial y}\right)\\ \underline{x}=\underline{\varphi }\left(\underline{X}\right)=\underline{X}+\underline{U}+\frac{\partial \underline{U}}{\partial \underline{X}}\left(\underline{X}-\underline{X_{0}}\right) \end{array},$$ (2)

where x designates the final configuration and X, the initial one.

The solution corresponding to the optimal kinematical transformation between initial and final configurations of a deformed specimen is computed by minimization of the correlation function S [Eq. (1)]. Initial values need to be calculated using a minimization process to ensure an optimization starts near the global solution.

To accurately measure the displacements in presence of a crack in the subset, the kinematical field was enriched by adding a Heaviside function $\;\underline{H}\prime$ (Valle et al., 2015) as shown in Eq. (3)

$$\underline{x}=\underline{\varphi }\left(\underline{X}\right)=\underline{X}+\underline{U}+\frac{\partial \underline{U}}{\partial \underline{X}}\left(\underline{X}-\underline{X_{0}}\right)+\underline{H}\prime \left(\underline{X}-\underline{X_{0}}\right).$$ (3)

**FIGURE 2:**
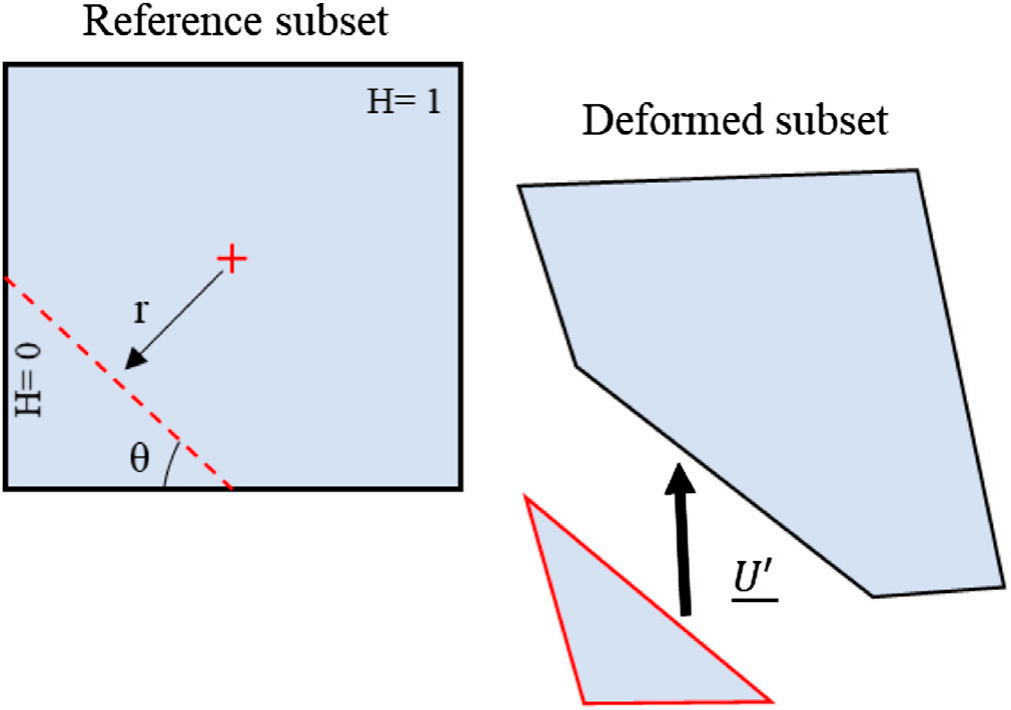
Heaviside function effect on a subset defining the discontinuity for the H-DIC process (Valle et al., 2015).

In the first part of the above representation, the terms of a Taylor development issued from the classical DIC can be retrieved, and the second part, which corresponds to the Heaviside term as illustrated in Figure 2, cuts the subset into two parts. This representation can be formulated as a classical DIC analysis on two separate sub-domains *D*_*1*_ and *D*_*2*_ as shown in Eq. (4)

$$\{\begin{array}{c} \varphi_{1}\left(\underline{X}\right)=\underline{X}+\underline{U_{1}}+\frac{\partial U}{\partial X}\left(\underline{X}-\underline{X_{0}}\right)on\;domain\;D_{1}\;\left(H=0\right)\;\;\\ \varphi_{2}\left(\underline{X}\right)=\underline{X}+\underline{U_{2}}+\frac{\partial U}{\partial X}\left(\underline{X}-\underline{X_{0}}\right)on\;domain\;D_{2}\;\left(H=1\right) \end{array},$$ (4)

where r and θ define the position and the orientation of the two-dimensional Heaviside function ${\underline{H}}^{\prime }$ .

The optimized solution S is formulated as shown in Eq. (5)

$$S\left(u,v,\;\frac{\partial u}{\partial x},\;\frac{\partial v}{\partial x},\;\frac{\partial u}{\partial y}\;,\;\frac{\partial v}{\partial y},\;u\prime ,\;v\prime ,\theta ,\;\;r\;\right),$$ (5)

with $\underline{U}\prime =\left(u\prime ,\;v\prime \right)$ the jump vector and $H\left(\underline{X}-\underline{X_{0}}\right)=H\left(r,\theta \right)$ defining a right line in a circular base and using the representation in Figure 2.

Parameters $\left(u,v,\;\frac{\partial u}{\partial x},\;\frac{\partial v}{\partial x},\;\frac{\partial u}{\partial y}\;,\;\frac{\partial v}{\partial y},\;u\prime ,\;v\prime ,\theta ,\;\;r\;\right)$ are optimized in a single process, and all subsets are enriched. When there is no discontinuity present in the subset, the optimization gives a jump $\underline{U}\prime$ near zero and automatically deactivates the Heaviside term.

The optimization process, based on a descending gradient algorithm, was employed to retrieve the displacement for each subset. Moreover, the algorithm implemented in “Massive Parallel Computation” with a GPU card allows high-speed and high-resolution analysis.

### 2.4 Finite Element (FE) Analysis

To obtain a better understanding of bone behavior when filled with PMMA cement and to dispose of stress analysis, we built a preliminary FE model from the comparison with the displacement and strain of experimental results. We used the same geometry we had in our samples without a balloon. The sample was discretized in two parts, a cement cylinder inside the cubic cancellous bone sample. The quality of the mesh was evaluated from a convergence study to verify the optimal mesh parameters. The resulting mesh was composed of four-noded tetrahedral elements (mean edge length, 0.5 mm). The experimental boundary conditions were mimicked as realistically as possible in the FE simulations (Table 2). The mechanical properties of materials are given in Table 3. The Young modulus of the cement (PMMA material) and of the cancellous bone was evaluated from experimental tests (Vendeuvre et al., 2019).

**TABLE 2:** Boundary conditions used for FE analysis.

Model 1	Model 2
Two materials bonded	No separation contacts without friction between two materials
Zero imposed displacement on the lower face	
Load applied on the upper face	

**TABLE 3:** Material properties used for FE analysis.

Property	Cancellous bone	PMMA
Young modulus (MPa)	150	2500
Poisson ratio	0.3	0.3

The FE simulations were performed using ANSYS^®^ software (release 16.1, ANSYS, Inc., United States ).2.5.

## 3 Results

### 3.1 Displacement-Load Curves

Individual results for the six cases, that is, cement/cancellous interface with a balloon and without a balloon with a strain rate of 0.001 mm/min, 0.05 mm/min, and 0.1 mm/min are presented here. The compression loading conditions (as shown in Figure 1) were chosen to mimic real-time loading of tibial fractures, especially for type I (split), II (split and depression), and type-III (depression) of Schatzker classification (Schatzker et al., 1979).

**FIGURE 3:**
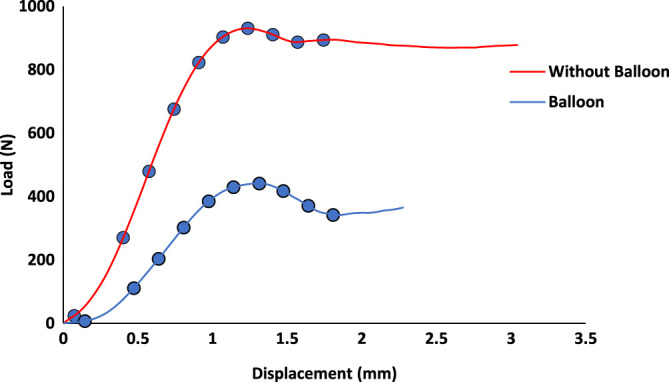
Load–displacement curve for a sample with a 1 mm/min loading condition, with and without a balloon. The circles on the curve represent the loading steps that were chosen to analyze using H-DIC.

A representative load vs. displacement response for the six bone specimens is shown in Figure 3. Incremental loading of the specimens was then continued until the final fracture and digital images were recorded to identify the incremental crack growth.

### 3.2 Displacement Field

**FIGURE 4:**
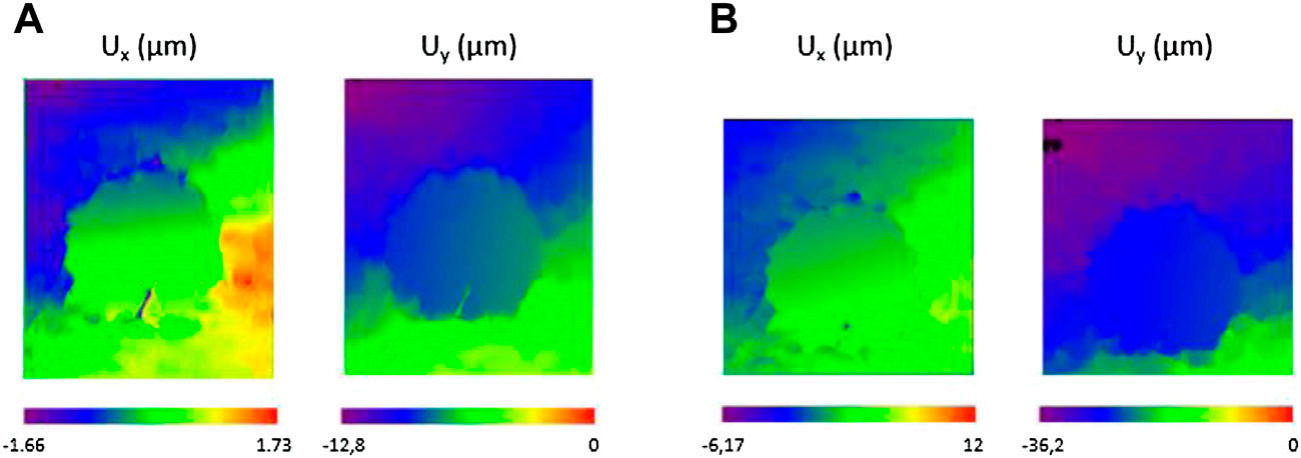
Examples of displacement fields for a sample without a balloon at **(A)** 0.66 mm and **(B)** 1.33 mm of imposed displacement (loading speed: 1 mm/min).

An image of the specimen was recorded prior to the loading (reference step) and at each loading step of the displacement (every 2 s during the loading). These images were later used for computing the displacement fields using the H-DIC method at every load step. Some illustrations of displacement fields are shown in Figure 4 from a few load steps. Analysis was conducted by H-DIC with a subset size of 48*48 pixels used for all the computations for identifying discontinuity and for measuring displacement.

With the proposed H-DIC method, the discontinuities were observed both near and far from the crack with variation in the gradient values throughout the boundaries in both *X* and *Y* directions. The enriched Heaviside function was very efficient at giving results for the zone influenced by fractures. The accuracy of the gradients in the horizontal *U*_*x*_ component and vertical *U*_*y*_ component were well retrieved in H-DIC. The displacement fields according to both directions show the absence of displacement inside the cement compared to the cancellous bone where there was the maximum displacement. The cement behaved like a rigid body inside a material that is far less stiff.

### 3.3 Strain Distribution and Crack Identification

**FIGURE 5:**
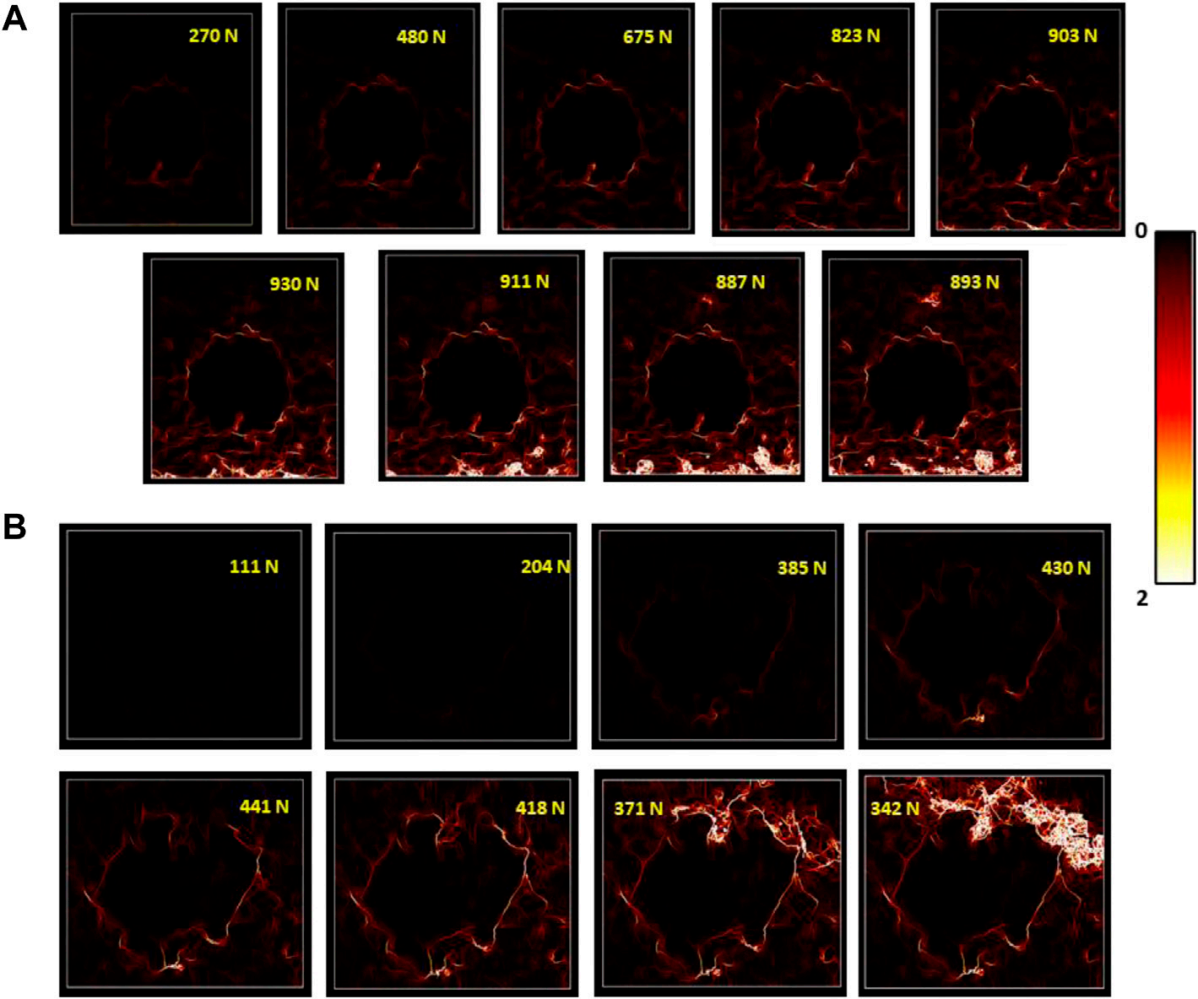
Evolution of equivalent strain fields obtained from the DIC method for consecutive loading steps for **(A)** sample without a balloon and **(B)** with a balloon.

Figure 5 shows the equivalent strain fields for the sample with and without a balloon for 1 mm/min at the bone/cement interface. For the same strain gradient value, successive loading steps were considered to identify the discontinuity from the initial state to the final fracture. The strain maps resulting from H-DIC demonstrated clear localization of the crack and reliable evolution of the µcrack until a final fracture was observed in the interface between cement and cancellous bone.

We see clearly in Figure 5 that the discontinuity has been initiated between bone and cement. Even if we were still were in the linear part of the curve (Figure 3), there were already discontinuities and even the beginning of cracks. So, there was a clear discontinuity between both materials which led to the degradation, when it occurs, of the cancellous bone alone.

**FIGURE 6:**
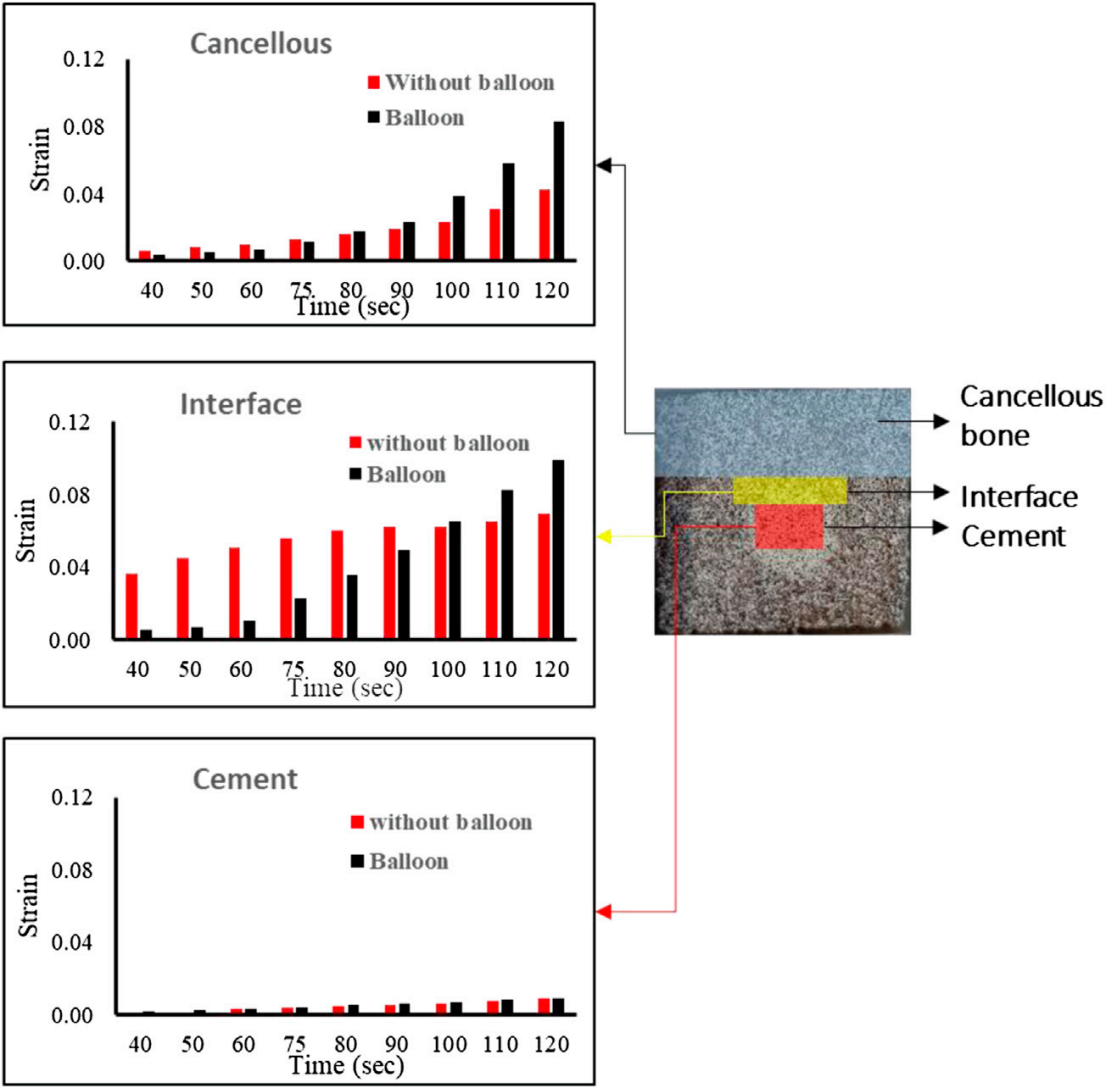
Strain variation in different zones for a sample without the balloon and with the balloon.

Figure 6 shows the equivalent strain in different localizations in the specimen. The two configurations were compared (with the balloon, in black, and without the balloon, in red). For cancellous bone, we had a similar strain initially and after the fracture. The strain increased abruptly in the specimen in both cases.

In cement, for both specimens, the strain was almost the same with a slight increase after the fracture. In the interface, in the specimen without a balloon, the strains were very high from the beginning and were almost constant with a slight increase after a fracture. However, in specimens with a balloon, the strain increased as the load increased even after the fracture.

### 3.4 Which Finite Element Model Matches the Experiment

**FIGURE 7:**
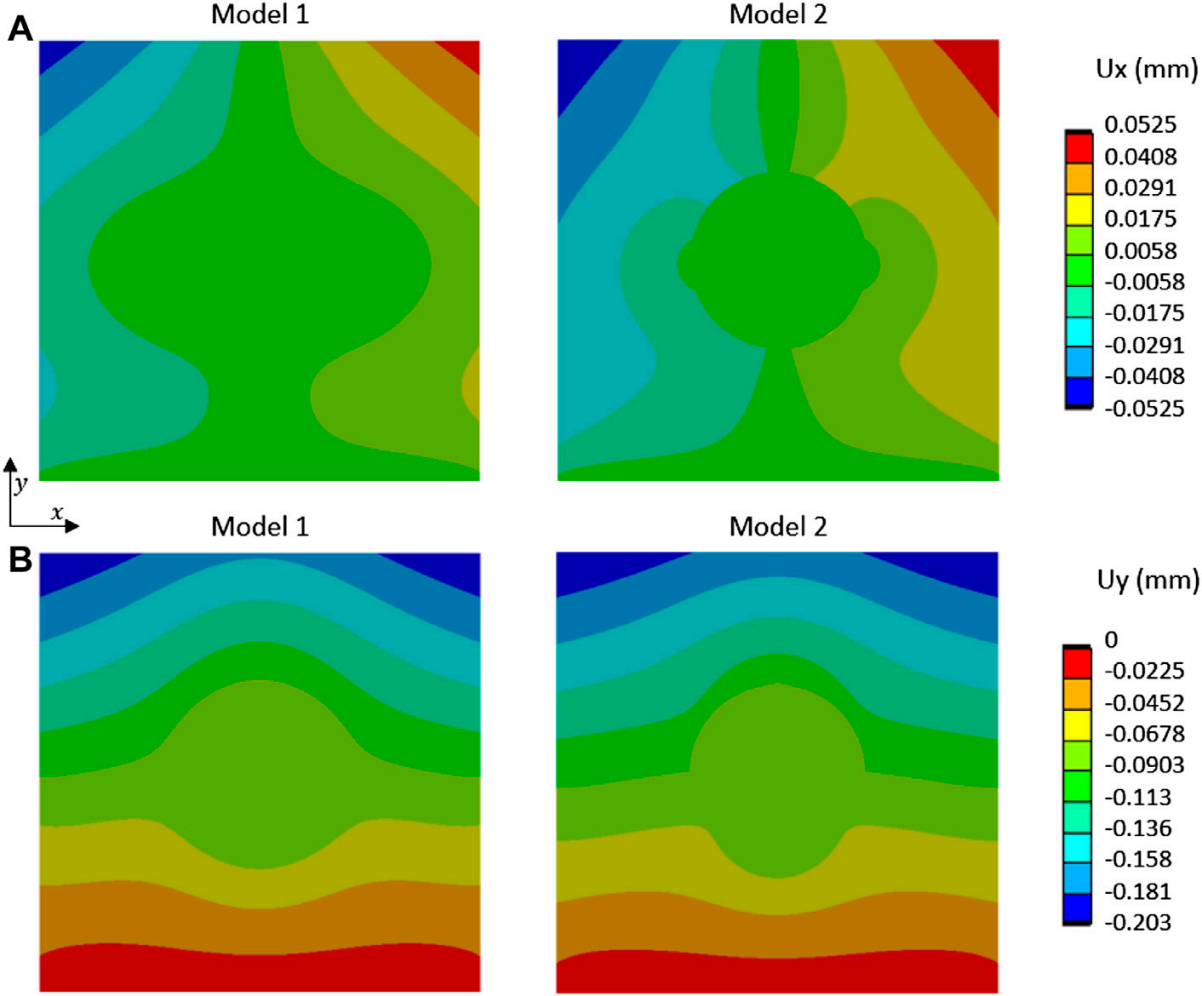
Displacement fields, **(A)** U_x_ and **(B)** U_y_, from FE simulation of compression tests for two models (Model 1: cement and cancellous bone were bonded; Model 2: cement and cancellous bone were not bonded without separation) and loading of 500 N.

The results of displacement fields from the two different models are shown in Figure 7 for a compression loading of 500 N. The most relevant model in terms of displacement and strain was the model without a link between the two materials. In this model, as in our experimentation, a discontinuity of mechanical behavior occurred at the interface, involving a slight heterogeneous displacement of the cement part.

## 4 Discussion

### 4.1 Mechanical Behavior of Cement/Bone Tissue Interface

The identification of mechanical fields and cracks in the cement/cancellous bone interface was important to understand the biomechanical behavior of the bone filled with cement in the treatment of tibial plateau fracture treated by tuberoplasty. No similar work, especially corresponding to the cement/cancellous interface fracture, has been reported earlier using digital image correlation. A clear evolution of the fracture line could be observed between the interfaces from the initial to the final state. It demonstrated the discontinuity between both materials that begin early but caused cracks and fracture after a linear behavior. This demonstration was carried out with the use of a novel correlation process: H-DIC. There is no equivalent method available in the literature to analyze bone tissues during continuous loading. With the help of H-DIC, it was possible to identify and localize fracture zone, multiple fracture lines, and multiple µcracks even at a very low strain gradient and with good accuracy.

### 4.2 Same Curves Without Cement but Crack Initiation and Propagation Identical to the Cancellous–Cortical Interface

**FIGURE 8:**
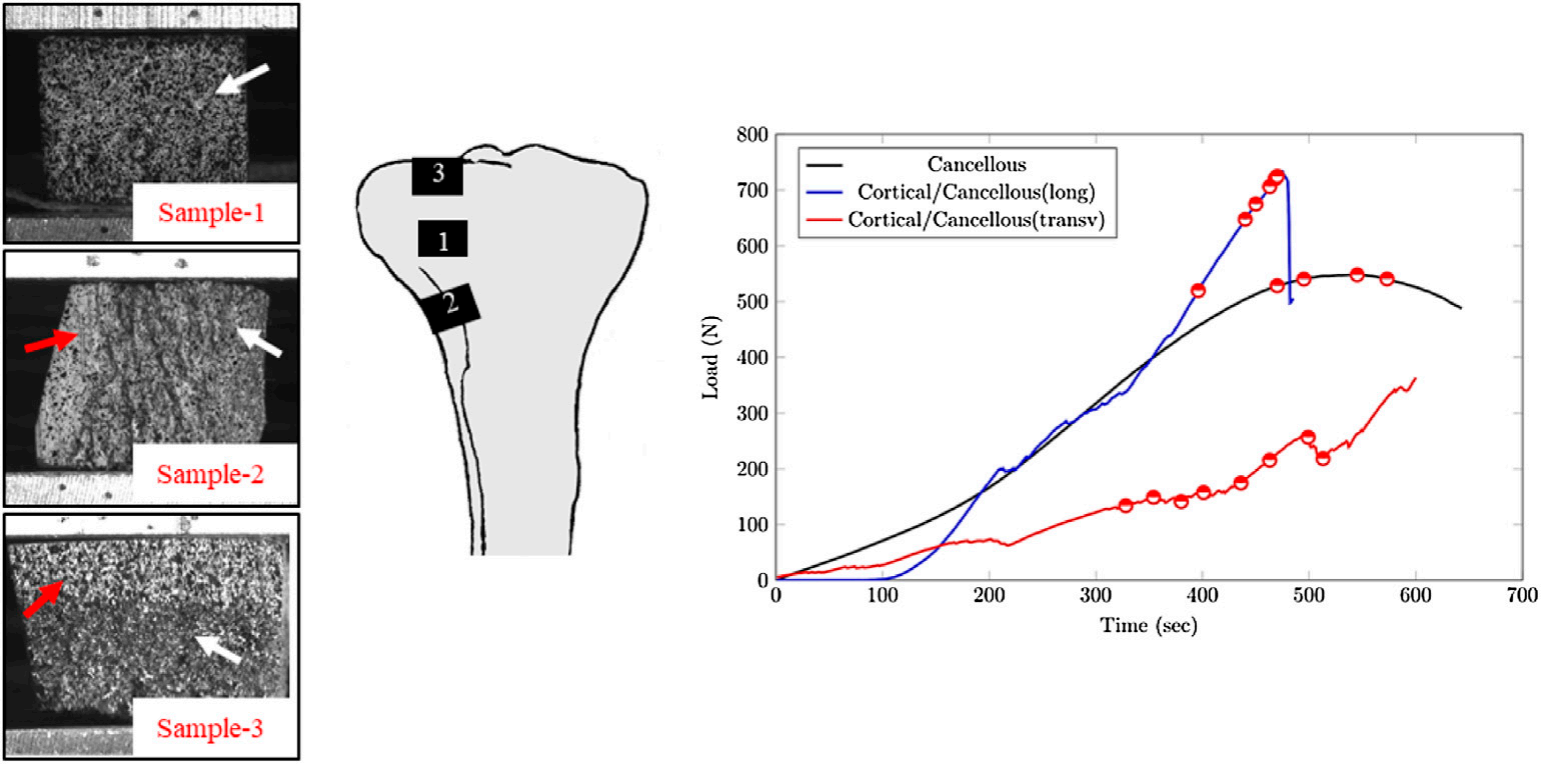
Load vs. time plot for different sample configurations: Sample 1—cancellous bone, Sample 2—cortical/cancellous in a longitudinal orientation, and Sample 3—tibial plateau in a transverse orientation.

In a recent work (Bokam et al., 2020), a compression test using H-DIC correlation showed the behavior of different configurations of bone without cement (Figure 8). The load vs. time plot is illustrated in Figure 8.

**FIGURE 9:**
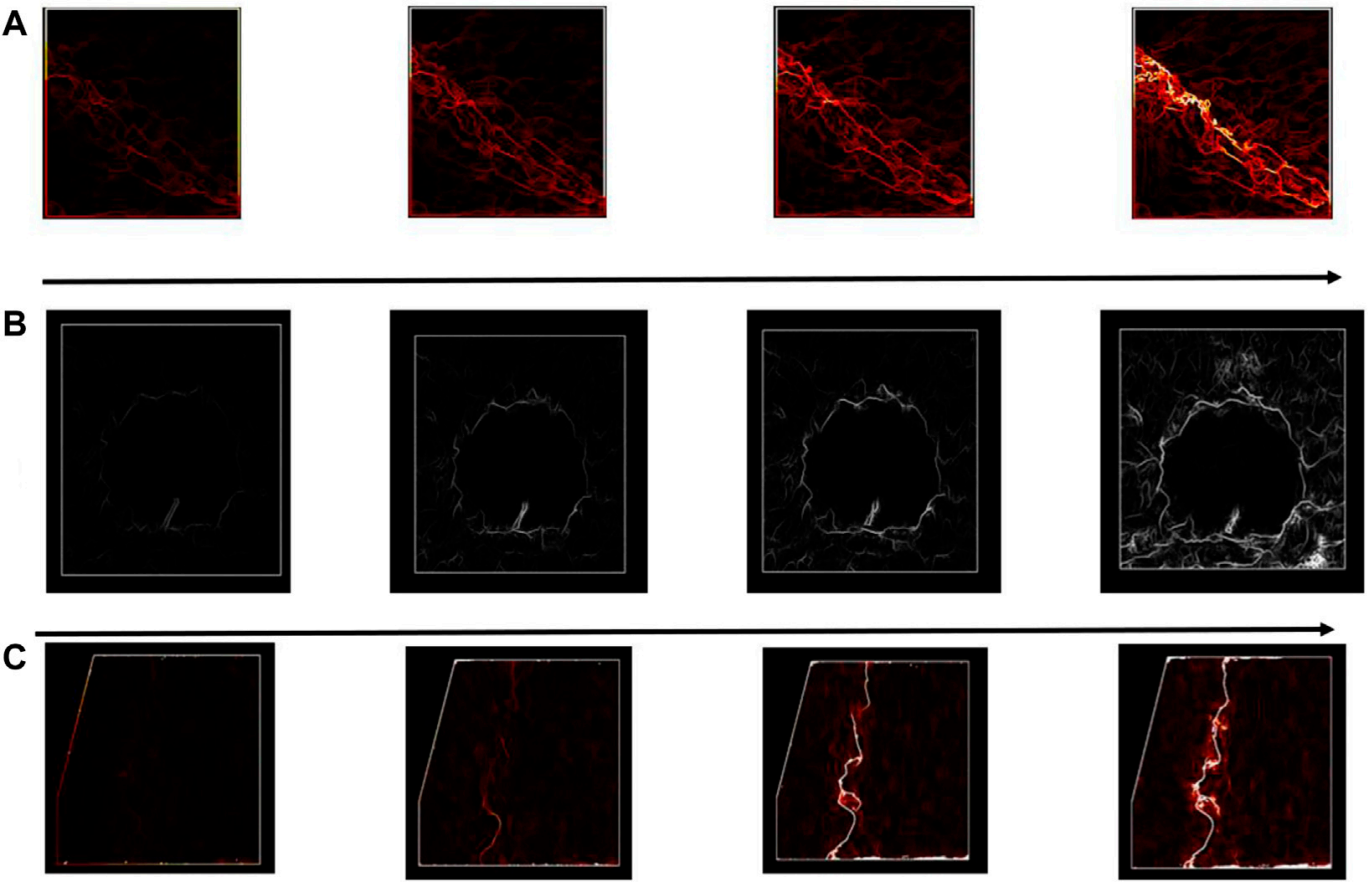
Equivalent strain displacement comparison for consecutive loading steps between different configurations for **(A)** cancellous bone, **(B)** cancellous–cement sample, and **(C)** cancellous–cortical samples, respectively.

In our present work, the load versus time plot is similar to the one of cancellous bone without cement and without cortical. However, the initiation and propagation of the cracks were more similar to the configuration of sample 3 with cancellous–cortical interface (Figure 9).

### 4.3 Speed and Balloon Contribution

During a tibial plateau fracture, there is a fracture in the cortical bone and compaction of the cancellous bone below. To reduce the fracture during tuberoplasty, a balloon is inflated, inducing compaction of the cancellous bone around the balloon (Vendeuvre et al., 2013; Belaid et al., 2018). To be as close to reality as possible, we decided to create a cavity using compaction due to the inflation of the balloon.

The results of our study showed no difference in the initiation of the cracks that occurred at the cement–bone interface, and no difference in the displacement map with no displacement in the cement. We obtained the same results of variation of the level of load needed to fail, to modify the speed of loading. These results are explained by a deterioration of the bone's mechanical properties by compacting the cancellous bone.

But the compaction induced a modification of bone density, and the contrast between cement and healthy cancellous was far lower which explained the results we show in Figure 7. With balloon and bone compaction, there was a transfer of strain carried out without changes of behavior at the interface compared to the group without a balloon. This explains the fact that in samples with a balloon, the discontinuity began later in the linear part of the curve. Whereas, without balloon samples, the first cracks which indicate the discontinuity appeared very early in the experiment, when we were still in the very beginning of the linear part.

### 4.4 Which Biomechanical Model and Clinical Relevance

**FIGURE 10:**
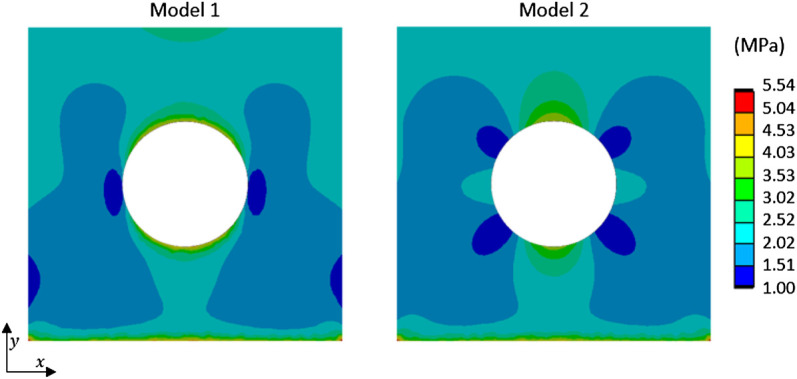
Equivalent stress fields from FE simulation of compression test for two models (Model 1: cement and cancellous bone were bonded; Model 2: cement and cancellous bone were not bonded without separation) and loading of 500 N.

Surgeons use the cement thinking that it will create a link with the different cancellous bone fragments (as in model 1 in Figure 7). They do not know if the mechanical properties of the cement permit weight-bearing on the operated foot. With the contact model used, the experiment demonstrated that the most relevant model would be model 2 (where cement and bone were not bonded). The FE approach provided stress analysis showing a higher stress concentration in the bone around the cement with model 2 (Figure 10). In future works, an evolutionary model with bonded contact with bone and cement, which could debond above some stress limits, is important to better answer this issue.

In addition, the load needed to obtain a crack, which in real life means a bone fracture, is far under the load observed in the knee for a 75 kg male person while walking (Bergmann et al., 2014). Our work demonstrated that for the cement to provide good reduction and primary stabilization (Vendeuvre et al., 2013; Belaid et al., 2018), permitting the patient to undergo rehabilitation with active and passive mobilization, no weight-bearing should be authorized while the bone is not consolidated. These results were very interesting because it differed from some works showing that the stabilization of spine fracture with kyphoplasty was sufficient to stabilize the fracture, permitting the patient to walk 12 h after the surgery without any brace (Germaneau et al., 2016).

Two parameters could play a role to improve adhesion between bone and cement: the pressure of injection and the present viscosity of the injection. During surgery, cement is injected with pressure and with high viscosity to limit the leakage. An experimental biomechanical study with the variation of these two parameters must be performed to provide significant inputs concerning the optimal way to inject the cement. In addition, in the future, FE analysis on the treatment of tibial plateau fracture using adequate biomechanical characteristics with no contact bonding between both materials should be made to study the linear behavior before discontinuity.

### 4.5 Limitations

Owing to the aforementioned results, the present study has some limitations. The experiments have been performed using 2D image analyses that only provide surface strain measurements. A volume approach with X-ray micro-computed tomography analysis to measure the displacement fields by H-Digital Volume Correlation (H-DVC) (Valle et al., 2018) could provide a better understanding of the microstructure mechanical behavior in bone tissues and particular volume crack detection between bone and cement. This will be the next step of the present work. On the other hand, DVC analyses were performed with a limited number of static load steps making crack apparition and crack propagation difficult. Therefore, a 2D analysis with H-DIC would be very interesting for detecting microcracks, crack initiation, and propagation for various loading conditions without interrupting the loading. 2D *in vitro* analysis also allows highlighting the relevant static load steps for future 3D analysis. Moreover, in the present study, only a macro-scale sample (cuboid shape) was used as a first test, whereas, in the future, more experiments on the whole bone sample should be performed to improve the finite element model of bone treatment by tuberoplasty. Concerning FE analysis, we considered two contact models: bonded and without friction. An evolutionary model with bonded contact between bone and cement which could debond above some stress limits could be future work to complete the local analysis of mechanical effects of cement injection.

## 5 Conclusion

In this work, experiments were performed to study the bone’s mechanical behavior when filled with PMMA cement. Several parameters have been studied by using a novel correlation process highlighting the biomechanical role of the cement inside the bone. This demonstrated that there is a discontinuity of load transfer between bone and cement. After the surgery, the cement behaves like a rigid body inside the cancellous bone (same as a screw or plate). With balloon inflation and bone compaction, there was a transfer of strain carried out without changes of behavior at the interface, compared to specimens without the balloon. FE analysis has been performed and compared to other experiments. This approach underlined a higher stress concentration when there is no link between bone and cement.

The cement provides good reduction and primary stabilization with its large contact area with the bone (minimally invasive approach and good stress distribution), permitting the patient to undergo rehabilitation with active and passive mobilization, but no weight-bearing should be authorized while the cortical bone is not consolidated or stabilized.

## Data Availability

The raw data supporting the conclusions of this article will be made available by the authors, without undue reservation.
